# Efficient genome editing in *Claviceps purpurea* using a CRISPR/Cas9 ribonucleoprotein method

**DOI:** 10.1016/j.synbio.2022.02.002

**Published:** 2022-02-16

**Authors:** Lu Yu, Meili Xiao, Zhihua Zhu, Yinmei Wang, Zhihua Zhou, Pingping Wang, Gen Zou

**Affiliations:** a CAS-Key Laboratory of Synthetic Biology, CAS Center for Excellence in Molecular Plant Sciences, Institute of Plant Physiology and Ecology, Chinese Academy of Sciences, 300 Fenglin Rd, Shanghai, 200032, China; b University of Chinese Academy of Sciences, Beijing, 100049, China; c Shanghai Key Laboratory of Agricultural Genetics and Breeding, Institute of Edible Fungi, Shanghai Academy of Agriculture Science, 1000 Jinqi Rd, Fengxian, Shanghai, 201403, China

**Keywords:** Ribonucleoprotein, Genome editing, Ergot alkaloids, Biosynthetic pathway, Homologous recombination

## Abstract

*Claviceps purpurea* produces many pharmacologically important ergot alkaloids (EAS), which are widely used to treat migraine and hypertension and to aid childbirth. Although an EAS biosynthetic cluster of *C*. *purpurea* has been discovered more than 20 years ago, the complete biosynthetic pathway of EAS has not been fully characterized until now. The main obstacle to elucidating this pathway and strain modification is the lack of efficient genome-editing tools for *C*. *purpurea*. The conventional gene manipulation method for *C*. *purpurea* relies on homologous recombination (HR), although the efficiency of HR in *C*. *purpurea* is very low (∼1–5%). Consequently, the disruption of target genes is laborious and time-consuming. Although CRISPR/Cas9 genome-editing methods based on *in vivo* Cas9 expression and gRNA transcription have been reported recently, their gene-disruption efficiency is still very low. Here, we developed an efficient genome-editing system in *C*. *purpurea* based on *in vitro* assembled CRISPR/Cas9 gRNA ribonucleoprotein complexes. As proof of principle, three target genes were efficiently knocked out using this CRISPR/Cas9 ribonucleoprotein complex-mediated HR system, with editing efficiencies ranging from 50% to 100%. Inactivation of the three genes, which are closely related to uridine biosynthesis (*ura5*), hypha morphology (*rac*), and EAS production (*easA*), resulted in a uridine auxotrophic mutant, a mutant with a drastically different phenotype in axenic culture, and a mutant that did not produce EAS, respectively. Our ribonucleoprotein-based genome-editing system has a great advantage over conventional and *in vivo* CRISPR/Cas9 methods for genome editing in *C*. *purpurea*, which will greatly facilitate elucidation of the EAS biosynthetic pathway and other future basic and applied research on *C*. *purpurea*.

## 1 Introduction

*Claviceps purpurea* is a phytopathogenic fungus that grows on cereals and forage grasses. It produces pharmacologically important ergot alkaloids (EAS), such as ergosine, ergocristine, ergotamine, and ergonovine, which have been used to treat migraine, hypertension, or to aid childbirth [1]. An EAS biosynthetic cluster of *C*. *purpurea* was first discovered in 1999 [2], and several genes in the cluster have been characterized functionally by gene disruption and analysis of the intermediates [[3], [4], [5], [6], [7]]. However, the P450 enzyme responsible for the formation of key precursor elymoclavine and the non-ribosomal peptide synthases (NRPS) for the biosyntheses of downstream ergosine and ergocristine remained elusive [5]. Candidate genes for the above miss enzymes have been proposed either within or beyond the EAS cluster, efficient gene disruption tools would largely benefit the characterization and validation of these candidates.

The conventional gene-manipulation method in *C*. *purpurea* incorporates selection markers into the genome via homologous recombination (HR); however, the efficiency of HR in *C*. *purpurea* is very low (1–5%) [[5], [8]]. The disruption of target genes is expensive and time-consuming. Therefore, it is necessary to develop an efficient, versatile genome editing tool for *C*. *purpurea*. NHEJ (non-homologous end joining)-deficient background (such as Δ*ku70*, Δ*kusA* and Δ*ligD*) strains are generally used to improve the HR efficiency [9]. Operation of key HR-relating genes (such as *Rad51*, *Rad52*, and *Mph1*) is an alternative strategy [10]. Moreover, longer flanking sequences of a selection marker also facilitate the higher HR efficiency [11]. Recently, the CRISPR/Cas9 system has been successfully engineered in both prokaryotes and eukaryotes [[12], [13], [14]] and has proved to be a feasible, efficient, versatile, genome-engineering tool. This system consists of a Cas9 nuclease and a functional guide RNA (gRNA) [15]. The gRNA recognizes the target sequence in the genome, and the Cas9 nuclease cleaves the sequence to generate double-strand DNA breaks (DSBs) [16]. Then, the cells repair these breaks via nonhomologous end-joining or HR using exogenous DNA (donor DNA), resulting in insertion or deletion mutagenesis at targeted genomic loci [17,18].

CRISPR/Cas9 systems can be established by either *in vivo* or *in vitro* strategies. In the *in vivo* strategy, cassettes expressing Cas9 and gRNA with appropriate promoters are introduced into cells to produce a functional Cas9/gRNA complex *in vivo* [19]. Recently, an approach for CRISPR/Cas9-mediated mutagenesis in *C*. *purpurea* using *in vivo* expression of Cas9 and gRNA was described [20]. However, this strategy does not work well, since the editing efficiency of CRISPR/Cas9-mediated homology-directed repair was only slightly better than HR-mediated knock-out of the *TrpE* gene (4/116 and 6/384, respectively). In the *in vitro* strategy, the purified Cas9 protein and *in vitro* transcript gRNA are assembled *in vitro* to generate a functional Cas9/gRNA ribonucleoprotein (RNP) and then transformed into cells for genome editing. This strategy has obvious advantages over the *in vivo* strategy, as assembly of the Cas9/gRNA complex is not limited by the amount or rate of Cas9 translation or gRNA transcription *in vivo*. The editing efficiency reached 100% in the filamentous fungi *Trichoderma reesei* [11,21,22] and *Cordyceps militaris* [23,24] after optimizing the RNP-based method.

Here, we developed an efficient CRISPR/Cas9-mediated genome-editing method for *C*. *purpurea* using the *in vitro* strategy. Three target genes, which are closely related to uridine biosynthesis (*ura5*, encoding orotate phosphoribosyl transferase catalyzing the transformation of orotate to OMP in the pyrimidine pathway; accession No. KAG6317199) [25], hypha morphology (*rac*, encoding small GTPase involved in polarity, sporulation and hyphal growth of *C. purpurea*; accession No. CAO82105) [26], and EAS production (*easA*, encoding chanoclavine-I aldehyde oxidoreductase catalyzing chanoclavine-I-aldehyde to form more-complex ergot alkaloids; accession No. Q6ZXC1) [27], were selected to verify the efficiency of this system. In the experiments, all three genes were efficiently knocked out by the CRISPR/Cas9-mediated HR.

## 2 Materials and methods

### 2.1 Strains and culture media

The strain *C. purpurea* 3.1003 was purchased from China General Microbiological Culture Collection Center (CGMCC, Beijing, China), which was grown on potato dextrose agar medium (200 g potato infusion, 20 g dextrose, 20 g agar to 1 L water). For the fermentation of *C. purpurea* strain, the mycelia were cultivated for 5 days in the seed medium TS (100 g/L sucrose, 10 g/L asparagine, 0.5 g/L KH_2_PO_4_, 0.3 g/L MgSO_4_·7H_2_O, 0.007 g/L FeSO_4_·7H_2_O, 0.006 g/L ZnSO_4_·7H_2_O, 0.1 g/L yeast extract, at pH 5.2), then transferred to the fermentation medium B (100 g/L sucrose, 10 g/L citric acid, 1 g/L Ca(NO_3_)_2_, 0.5 g/L KH_2_PO_4_, 0.25 g/L MgSO_4_·7H_2_O, 0.007 g/L FeSO_4_·7H_2_O, 0.006 g/L ZnSO_4_·7H_2_O, 0.12 g/L KCl, at pH 5.2) and cultivated in the dark at 24 °C on a rotary shaker (200 rpm) for 4 weeks. *C. purpurea* was grown at 28 °C either for 3 days in CD medium [11] for protoplast preparation, or for 2 days in SDB medium (40 g/L dextrose, 10 g/L yeast extract, 10 g/L tryptone) for genomic DNA isolation. Protoplast regeneration medium was used as described by Zou et al. (2020) [11]. *Escherichia coli* Top10 used for gene cloning was cultured at 37 °C in Luria Bertani (LB) medium with ampicillin (100 μg/mL). Primers used in this study are listed in Table S2.

### 2.2 Preparation of target gene, Cas9 protein and gRNAs

Cas9 protein tagged with a nuclear localization signal was purchased from Novoprotein, Inc. (Shanghai, China). Since the genomic information of *C. purpurea* 3.1003 has not been reported yet, primers were designed to amplify the target genes using *C. purpurea* 20.1 as a reference. *In vitro* transcription of gRNA was carried out as previously described by Zou et al. [11]. The *in vitro* RNA product was incubated with Cas9 at 37 °C for 15 min to form a Cas9-gRNA complex before transformation into protoplasts. The assembling reaction system contained 6 μL Cas9 (1 μg/μL), 30 μL 10 × Cas9 activity buffer (0.2 M HEPES, 1.5 M KCl, 5.0 mM dithiothreitol, 1.0 mM EDTA, 0.1 M MgCl_2_, pH 7.5), 5 μL gRNA (∼3 μg/μL) and 46 μL 2 × S2 solution (2 M sorbitol, 100 mM CaCl_2_, 20 mM Tris-HCl, pH 7.5).

### 2.3 Protoplast preparation, transformation and selection

Mature conidia of *C. purpurea* were collected with 0.85% NaCl-0.02% Tween 80 and inoculated in 100 mL CD liquid medium for 3 days at 28 °C. The mycelia were then collected by filtration, and washed with solution 1 (1.2 M sorbitol, 0.1 M KH_2_PO_4_, pH 5.5). After that, the mycelia were suspended in 20 mL solution 1 with 12.5 mg/L lysing enzymes from *Trichoderma harzianum* (Sigma-Aldrich, St. Louis, MO, USA) and incubated at 28 °C for 2 h by mild agitation (50 rpm). The protoplasts were then separated from mycelia by filtration through sterilized Miracloth (Sigma-Aldrich). Then they were gently precipitated by centrifugation (2000 × *g*, 10 min, 4 °C) and washed once again with 4 mL solution 2 (1 M sorbitol, 50 mM CaCl_2_, 10 mM Tris-HCl, pH 7.5). Finally, the protoplasts were resuspended in solution 2 to a concentration of 10^7^/mL for subsequent transformation. Protoplasts release was checked by microscopic observation. The Cas9-gRNA complex and donor DNA were added into 200 μL of the above protoplast suspension. The aliquot was incubated on ice for 20 min and then solution 3 (2 mL) was added to the aliquot. After 30 min of incubation at 25 °C, solution 2 (4 mL) was added to the mixture. The resulting mixture was then poured into 50 mL melted regeneration medium containing 1.5 mg/mL hygromycin B and divided into three plates. After 7 days cultivation at 28 °C, transformants were inoculated onto a 24-well PDA plate (with 0.5 mg/mL hygromycin B). Colonies were picked and cultured in SDB for genomic DNA extraction. Gene disruption was validated by PCR amplification with primer pairs flanking the target site. All the sequence analyses of transformants were carried out by Sangon Biotech (Shanghai, China).

### 2.4 Construction of donor DNAs

The donor DNAs containing the 5′ and 3′ flanking sequences of target gene and the selectable marker cassette (Ptrpc-*hph*-Ttrpc) were generated by overlapping PCR using I5 DNA polymerase (Tsingke, Beijing, China) and ligated into the pMD-18T vector (Takara, Tokyo, Japan). The generated vector was propagated in *E. coli* Top10 and purified using the Plasmid Midi Kit (Qiagen, Hilden, Germany).

### 2.5 Analysis of ergot alkaloids

For alkaloid extraction and determination, cultures were extracted twice with ethyl acetate, and concentrated for further alkaloid analysis by HPLC. An Agilent 1100 HPLC system with a RP18-column (5 μm particle diameter, 250 × 4.6 mm) was used to separate and detect the alkaloids. The flow rate was 0.8 mL/min with a gradient elution of 15–95% (v/v) acetonitrile in 10 mM ammonium carbonate for 50 min. Detection was at 320 nm. Authentic ergocristine was purchased from Sigma–Aldrich Analyticals (Taufkirchen, Germany).

## 3 Results and discussion

### 3.1 Establishing genetic transformation and *in vitro* assembled Cas9/gRNA complex for *C*. *purpurea*

**Fig. 1:**
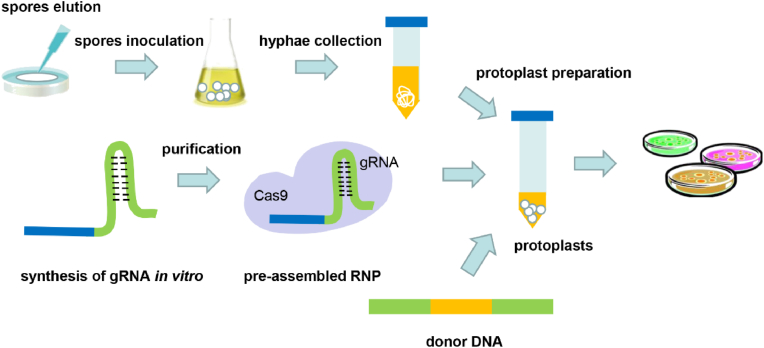
Overview of the RNP-based genome-editing system in *C*. *purpurea* in this study. Mature *C*. *purpurea* conidia were collected using 0.85% NaCl-0.02% Tween 80 and inoculated into 100 mL CD liquid medium. After 3 days, mycelia were collected and then suspended in solution 1 containing lysing enzymes to prepare protoplasts. Protoplasts were resuspended in solution 2 to a concentration of 10^7^/mL for subsequent transformation. RNA prepared *in vitro* was incubated with Cas9 at 37 °C for 15 min to form a Cas9/gRNA RNP complex. This complex was transformed into protoplasts with donor DNA. Finally, the resulting mixture was poured into 50 mL melted regeneration medium containing 1.5 mg/mL hygromycin B and divided into three plates.

The PEG-mediated protoplast transformation method was used to establish the genetic transformation system (Fig. 1). *C*. *purpurea* protoplasts were collected based on the preparation method used in *Aspergillus oryzae* with some modifications [11]. As shown in Fig. S1, approximately 10^7^ protoplasts/mL were obtained after incubating mycelia with 12.5 mg/mL lysing enzyme for 2 h, which is sufficient for a transformation experiment. The efficiency of this genetic transformation system was confirmed by transferring the Ptrpc-ble-Ttrpc fragment into the prepared protoplasts. After culturing at 28 °C for 7 days, monophyletic colonies were observed in the regeneration medium (containing phleomycin 30, 60, and 120 μg/mL) (Fig. S2), indicating that the Ptrpc-ble-Ttrpc fragment was successfully inserted into the *C*. *purpurea* genome, as confirmed by diagnostic PCR (Fig. S3).

To identify a resistance marker for genetic manipulation, phleomycin was primarily used as a selection agent based on a previous study in *C*. *purpurea* [5,8]. The resistance of *C*. *purpurea* to phleomycin increased with the pH of the culture medium. On pH 6.0 plates, phleomycin at 40–200 μg/mL lacked toxic effects, while phleomycin had strong restraining effects on a pH 8.0 plate containing 40 μg/mL phleomycin (Fig. S4). However, a high pH was not beneficial for the regeneration of *C*. *purpurea* protoplasts. Therefore, hygromycin, which is commonly used for genetic manipulation of fungi, was tested. After culturing at 28 °C for 27 days, mycelia growth was completely inhibited at a minimum inhibitory concentration of 0.5 mg/mL hygromycin on PDA plates (pH 6.0), which was used in the subsequent experiments (Fig. S5).

To develop an *in vitro* strategy for efficient genome editing in *C*. *purpurea* based on CRISPR/Cas9 technology, gRNAs for the target genes were transcribed *in vitro* via runoff reactions using T7 RNA polymerase. To improve the editing efficiency, gRNAs were designed for each target gene. Then, the *in vitro* RNA products were incubated with Cas9 protein to generate Cas9/gRNA complexes, according to our previously established CRISPR/Cas9 genome editing approaches for several fungi [11]. Next, the complexes were transformed into *C*. *purpurea* via PEG-mediated protoplast transformation (Fig. 1).

### 3.2 Gene disruption using the RNP-based CRISPR/Cas9 system in *C*. *purpurea*

**Fig. 2:**
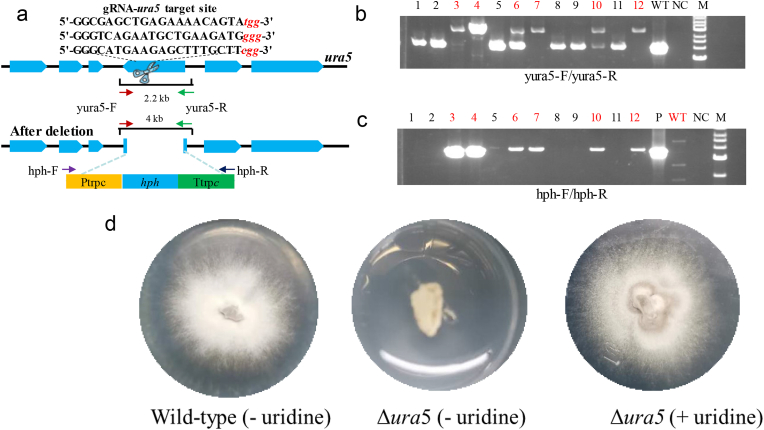
Knock-out of *ura5* in *C*. *purpurea* by CRISPR/Cas9-mediated homologous recombination (HR). (a) The knock-out strategy for the *ura5* gene. (b) Diagnostic PCR using primers yUra5-F and yUra5-R. (c) Diagnostic PCR using primers hph-F and hph-R. (d) The phenotypes of *C*. *purpurea* and the *ura5*-deficient mutant on CD plates with or without uridine cultured for 4 weeks at 28 °C. WT, wild-type; NC, negative control (using water as the amplification template); P: plasmid containing hygromycin selectable marker; M, marker.

A previous study used an HR strategy for genome editing in *C*. *purpurea*. However, HR was inefficient (9 positive transformants of 181 selected) [5]. Here, we first tested the efficiency of the ribonucleoprotein-based CRISPR/Cas9 system to stimulate HR using exogenous DNA (donor DNA) in *C*. *purpurea* by targeting the *ura5* gene encoding orotate phosphoribosyl transferase; this enzyme converts 5-fluoroorotic acid (5-FOA) into a toxic compound that severely inhibits the growth of wild fungal strains on medium. By contrast, the *ura5*-disruption mutant survives on plates with additional 5-FOA and uridine in the medium [11]. Three gRNAs were designed to target *ura5* (Fig. 2a) and were then incubated with Cas9 protein to generate Cas9/gRNA complexes. After co-transformation of the Cas9/gRNA complexes and donor DNA (containing the *hph* expression cassette) into *C*. *purpurea*, 12 transformants were obtained by hygromycin screening. Successful disruption of the *ura5* gene was indicated by a 4 kb PCR fragment produced using primers yUra5-F and yUra5-R and confirmed by sequencing the PCR product. Of the 12 colonies checked, 6 ones showed correct homologous integration (Fig. 2b), representing an editing efficiency of 50%. The PCR sequence from the remaining 6 obtained colonies demonstrated that the hygromycin selection marker was not incorporated into these colonies (Fig. 2c) as well as no mutations were found in all of the three targeted regions of their *ura5* genes (Fig. S6). These results indicated that we obtained half true colonies with the expected homologous recombination and half false positive colonies. The phenotypic study showed that the *ura5*-disruption mutant cannot survive on CD plates without uridine (Fig. 2d). Moreover, the uridine auxotrophic mutant could survive on PDA plates supplemented with 0.3 mg/mL 5-FOA and 10 mM uridine, while the wild-type showed no 5-FOA resistance (Fig. S7).

The *ura5* gene has been used as an effective selection marker for genome editing in fungi [11,28]. As there are few selection markers for genome editing in *C*. *purpurea*, the *ura5* gene may serve as an additional selectable marker for genome engineering in *C*. *purpurea*.

**Fig. 3:**
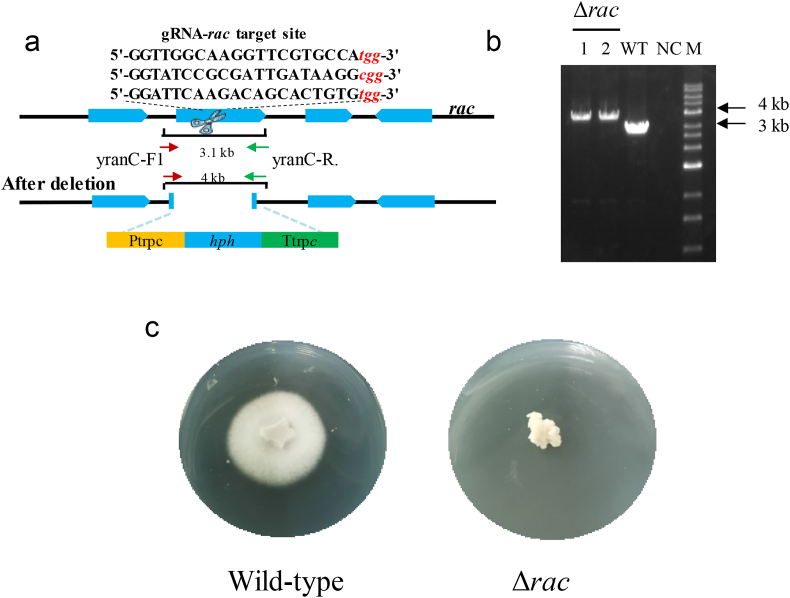
Knock-out of *rac* in *C*. *purpurea* by CRISPR/Cas9-mediated homologous recombination (HR). (a) The knock-out strategy for the *rac* gene. (b) Diagnostic PCR using primers yranC-F1 and yranC-R. (c) The phenotypes of *C*. *purpurea* and the Δ*rac* mutant on PDA plates. WT, wild-type; NC, negative control (using water as the amplification template); M, marker.

We subsequently tested the efficiency of the ribonucleoprotein-based CRISPR/Cas9 system at stimulating HR in *C*. *purpurea* by targeting the *rac* gene, which strongly influences the morphology of *C*. *purpurea* hyphae [26]. Three gRNAs were also designed to target *rac* (Fig. 3a). After co-transformation of the Cas9/gRNA complexes and donor DNA (containing the *hph* expression cassette) into *C*. *purpurea*, only two transformants were obtained by hygromycin screening. Fortunately, both were positive mutants, as confirmed by diagnostic PCR and Sanger sequencing (Fig. 3b). The *rac*-disruption mutants had a drastically different phenotype in axenic culture compared with the wild-type; they had a convoluted three-dimensional coralline form (without invading the agar), while the wild-type had a normal flat, two-dimensional form (Fig. 3c), as reported previously [26].

### 3.3 Using the RNP-based genome-editing system to confirm that a key enzyme participates in EAS synthesis in *C*. *purpurea*

**Fig. 4:**
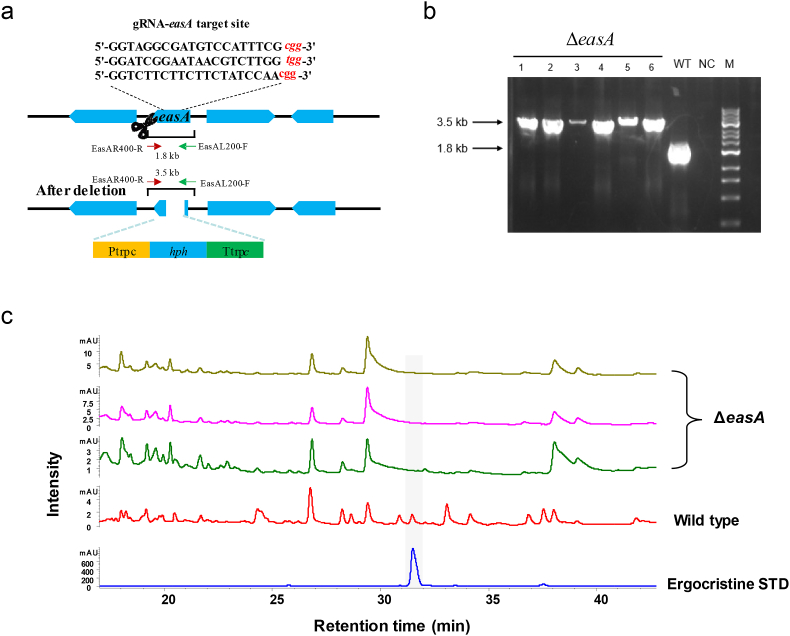
Knock-out of *easA* in *C*. *purpurea* by CRISPR/Cas9-mediated homologous recombination (HR). (a) The knock-out strategy for the *easA* gene. (b) Diagnostic PCR using primers EasAL200-F and EasAR400-R. (c) Analysis of the fermentation products of *C*. *purpurea* and the Δ*easA* mutant. Three Δ*easA* transformants were tested. WT, wild-type; NC, negative control (using water as the amplification template); M, marker.

The synthesis of clavines and lysergic acid derivatives starts with the formation of dimethylallyltryptophan (DMAT) from tryptophan and dimethylallylpyrophosphate (DMAPP) catalyzed by the first specific enzyme, i.e., dimethylallyltryptophan synthase (DMATS, coded by *dmaW*). Then, EasF methylates DMAT into MeDMAT. The generated MeDMAT is catalyzed by chanoclavine synthases (*easC* and *easE*) to form tetracylic ergolene, chanoclavine-I. In the presence of EasD, the chanoclavine-I aldehyde is generated. The *easA* gene was identified to encode chanoclavine-I aldehyde oxidoreductase. Through the catalysis of EasG, agroclavine is produced. However, the P450 for the production of elymoclavine has yet been defined in the EAS pathway. Another P450 (*CloA*) is responsible for paspalic acid producing. The following process is controversial. Some researchers believe that paspalic acid spontaneously isomerizes to d-lysergic acid. However, some researchers believe that it is catalyzed by an unknown enzyme [27]. In ergopeptine-producing ergot fungi, d-lysergic acid is assembled into the corresponding D-lysergyl peptides by the action of NRPS called lysergyl peptide synthetase 1 and 2 (LPS1 and 2). Among the EAS biosynthetic cluster, *easA* is a confirmed essential gene for EAS synthesis. Using the knock-out strategy shown in Fig. 4a, the partial *easA* ORF was replaced by an integrated *hph* expression cassette. After protoplast transformation, six transformants were obtained on PDA plates with hygromycin B. These transformants were tested by PCR using the primers easA-F/R, and all six transformants produced a 3.5 kb fragment (Fig. 4b), demonstrating that the *hph* cassette replaced parts of the *easA* gene. DNA sequencing of the PCR products confirmed that the targeted partial ORF of *easA* had been knocked out. The editing efficiency of HR and CRISPR/Cas9-mediated HR was compared by introducing dDNA-*easA* (containing the Ptrpc-*hph*-Ttrpc cassette and the 3ʹ and 5ʹ flanking regions of *easA*) into *C*. *purpurea* protoplasts. No transformants were obtained by the direct HR approach, indicating that our CRISPR/Cas9 system is an efficient, versatile genome-editing tool for *C*. *purpurea*. The *easA* gene encodes a key enzyme that is thought to take part in the EAS biosynthetic pathway in *C*. *purpurea* (Fig. S8) [5]. Disruption of this gene may lead to biosynthetic interruption of the downstream EAS, of which ergocristine is an end product (in red in Fig. S8). Thus, we checked whether the Δ*easA* mutants and wild-type produced ergocristine. The wild-type and *easA* mutant *C*. *purpurea* strains were fermented for 4 weeks, and the fermentation broths were extracted with ethyl acetate to analyze the EAS products. HPLC analysis detected no ergocristine in the fermentation extracts of the Δ*easA* mutants, while it was detected in the fermentation extracts of the wild-type strain (Figs. 4c and S9). These results indicate that inactivation of *easA* abolished EAS production in *C*. *purpurea*.

In summary, our CRISPR/Cas9 system based on *in vitro* assembled Cas9/gRNA RNP effectively stimulates HR in *C*. *purpurea*. Compared with the *in vivo* strategy in *C*. *purpurea*, this system has obvious advantages in terms of the process and editing efficiency (Table 1). Moreover, for those strains lacking genomic information, such as *C*. *purpurea* 3.1003 used here, it is difficult to establish a CRISPR/Cas9 system via an *in vivo* strategy, since neither codon-optimized *Cas9* genes nor RNA polymerase III promoters can be produced without genomic information. The RNP-based method established here overcomes these difficulties via *in vitro* transcription of gRNA and purification of Cas9 protein. Because of the transient existence of the RNP complex in cells, *in vitro* assembled and delivered Cas9/gRNA-based genome editing reduces the risk of unnecessary gene targeting and avoids the integrated mutations that may result from gene-delivery strategies [[29], [30], [31]].

**Table 1:** Comparison of *in vivo* and *in vitro* CRISPR/Cas9 strategies.

Strategy	Strain	Genome information	Target gene	HR vs NHEJ	Expression of Cas9 and gRNA	Reference
Plasmids	*C. purpurea* 20.1	Reported	*Trpe*	4/116 (3.4%)	Expression of Cas9 and gRNA based on transforming plasmids with gRNA and codon-optimized Cas9 gene i*n vivo*	[15]
RNPs	*C. purpurea* 3.1003	*None*	*easA*	6/6 (100%)	Unrestricted synthesis/expression of Cas9 and gRNA for pre-assembling RNPs *in vitro*	This study

In conclusion, we developed a simple, efficient CRISPR/Cas9 system in *C*. *purpurea* based on gRNA *in vitro* transcription. Target genes were efficiently disrupted by CRISPR/Cas9-mediated HR. Based on genomic information, the candidate genes involved in EAS pathway could be deleted efficiently using our developed method. With the advantage of counter selection using 5-FOA, *ura5* could be utilized as the bidirectional selection marker for recyclable genome editing. These strategies rapidly achieved simultaneous or stepwise deletion of multiple candidate genes. This work will be useful for metabolic engineering and gene regulation of *C*. *purpurea*, as well as elucidating the EAS pathway.

## CRediT authorship contribution statement

**Lu Yu:** Conceptualization, Investigation, Writing – original draft. **Meili Xiao:** Conceptualization, Investigation, Writing – original draft. **Zhihua Zhu:** Investigation. **Yinmei Wang:** Investigation. **Zhihua Zhou:** Project administration, Conceptualization, Supervision. **Pingping Wang:** Conceptualization, Writing – review & editing, Supervision. **Gen Zou:** Conceptualization, Writing – review & editing, Supervision.

## Declaration of competing interest

The authors have no interests to declare.
