# Supplementary material for: Efficient genome editing in Claviceps purpurea using a CRISPR/Cas9 ribonucleoprotein method
Source: Synth Syst Biotechnol. 2022 Feb 16;7(2):664–70. doi: 10.1016/j.synbio.2022.02.002 (PMC8857428; doi:10.1016/j.synbio.2022.02.002)
Supplement: Multimedia component 1 [file mmc1.doc]

Supplementary Information for:

**Efficient genome editing in *Claviceps purpurea* using a CRISPR/Cas9 ribonucleoprotein method**

Lu Yu et al.

**Table S1** Oligonucleotides and gene sequences used in this study.

**Fig. S1** Microscopic observation of the protoplasts of *C. purpurea* 3.1003 prepared.

**Fig. S2** Protoplast regeneration of *C. purpurea* 3.1003 under different concentrations of phleomycin.

**Fig. S3** Diagnostic PCR validation of transformants picked out under different stress of phleomycin.

**Fig. S4** Resistance sensitivity of *C. purpurea* 3.1003 to phleomycin on plates with various pH values.

**Fig. S5** Resistance of *C. purpurea* 3.1003 to hygromycin on PDA plates.

**Fig. S6** Sequences of ura5 gene of the false positive colonies.

**Fig. S7** Phenotype of *C. purpurea* and *ura5*-deficient mutant on PDA plates with 0.3 mg/mL 5-FOA. Cultivation was done for 4 weeks at 28℃.

**Fig. S8** Putative biosynthetic pathway of ergot alkaloids in *C. purpurea*. The dashed arrow indicates an unresolved step, and the solid-line arrows indicate that the step has been resolved. Ergot alkaloids catalyzed by *easA* in the pathway are marked in red.

**Fig. S9** Analysis of the fermentation products of *C. purpurea* and the Δ*easA* mutant. Three Δ*easA* transformants were tested. Black dotted line region of the HPLC spectrum was zoomed in to show detailed information.

**Table S1** Primers used in this study

| Name | Sequence(5’to3’) | Experiment |
| --- | --- | --- |
| EasA_gRNA1F | TAATACGACTCACTATAGGTAGGCGATGTCCATTTCGGTTTTAGAGCTAGAAATAGCAA | Amplification of DNA template of *easA*-gRNA1 |
| EasA_gRNA2F | TAATACGACTCACTATAGGATCGGAATAACGTCTTGGGTTTTAGAGCTAGAAATAGCAA | Amplification of DNA template of *easA*-gRNA2 |
| EasA_gRNA3F | TAATACGACTCACTATAGGTCTTCTTCTTCTATCCAAGTTTTAGAGCTAGAAATAGCAA | Amplification of DNA template of *easA*-gRNA3 |
| URA5-gRNA1F | TAATACGACTCACTATAGGCGAGCTGAGAAAACAGTAGTTTTAGAGCTAGAAATAGCAA | Amplification of DNA template of *ura5*-gRNA1 |
| URA5-gRNA2F | TAATACGACTCACTATAGGGTCAGAATGCTGAAGATGGTTTTAGAGCTAGAAATAGCAA | Amplification of DNA template of *ura5*-gRNA2 |
| URA5-gRNA3F | TAATACGACTCACTATAGGGCATGAAGAGCTTTGCTTGTTTTAGAGCTAGAAATAGCAA | Amplification of DNA template of *ura5*-gRNA3 |
| Rac_gRNA1F | TAATACGACTCACTATAGGTTGGCAAGGTTCGTGCCAGTTTTAGAGCTAGAAATAGCAA | Amplification of DNA template of *rac*-gRNA1 |
| Rac_gRNA2F | TAATACGACTCACTATAGGTATCCGCGATTGATAAGGGTTTTAGAGCTAGAAATAGCAA | Amplification of DNA template of *rac*-gRNA2 |
| Rac_gRNA3F | TAATACGACTCACTATAGGATTCAAGACAGCACTGTGGTTTTAGAGCTAGAAATAGCAA | Amplification of DNA template of *rac*-gRNA3 |
| gRNA R | AAAAGCACCGACTCGGTGCCACTTTTTCAAGTTGATAACGGACTAGCCTTATTTTAACTTGCTATTTCTAGCTCTAAA | Amplification of DNA template of *easA*-gRNA, *ura5*-gRNA, *rac*-gRNA |
| BW_CP(EasA)_gRNA_5’F | ATGCCTGCAGGTCGACGATtCAACCACAAGGTTGTGCTCT | Cloning the 5’ flanking region of *easA* |
| BW_CP(EasA)_5’R(hph) | TTAGTGAGGGTTAATTGCGCCAAACTGAGGAACTAGGTCG | Cloning the 5’ flanking region of *easA* |
| BW_CP(EasA)_3’F(hph) | AAATTCCGTCACCAGCCCTGACATCTCCAATCCGGATCTT | Cloning the 3’ flanking region of *easA* |
| BW_CP(EasA)_gRNA_3’R | CCCGGGGATCCTCTAGAGATCCGTCTGGTCTAAATATTCT | Cloning the 3’ flanking region of *easA* |
| EasAL200-F | CCGCCAAGAAGTTGAGCGAAGGG | Verifying the*△easA* transformant |
| EasAR400-R | CCCGGCGGATGCGTTTGAGA | Verifying the*△easA* transformant |
| Hph-F | GCGCAATTAACCCTCACTAAAGGGAA | Cloning of *hph* cassette |
| Hph-R | CAGGGCTGGTGACGGAATTTTCATA | Cloning of *hph* cassette |
| Ura5-5’F(764bp) | ATGCCTGCAGGTCGACGATtACTTCCTAGAGTGGCCCCGC | Cloning the 5’ flanking region of *ura5* |
| Ura5-5’R(hph) | TTAGTGAGGGTTAATTGCGCCGGCGGCAGGCAGCTTCTCC | Cloning the 5’ flanking region of *ura5* |
| Ura5-3’F(hph) | AAATTCCGTCACCAGCCCTGAAGTACAAGGCGTCTGACTA | Cloning the 3’ flanking region of *ura5* |
| Ura5-3’R(1031bp) | CCCGGGGATCCTCTAGAGATGTAGAGACCAGTAGGACGGA | Cloning the 3’ flanking region of *ura5* |
| yUra5-F | GCAGAAAGAAGCCTGAACGTCCGAT | Verifying the*△ura5* transformant |
| yUra5-R | GTCTTGCAGCGCTGAACAGGTATACTCA | Verifying the*△ura5* transformant |
| 5’F | ATGCCTGCAGGTCGACGATtCAGTACAGGTACCTATGTAC | Cloning the 5’ flanking region of *rac* |
| 5’R | TTAGTGAGGGTTAATTGCGCGTCAGCGCAAGAACAAGATG | Cloning the 5’ flanking region of *rac* |
| 3’F | AAATTCCGTCACCAGCCCTGATGATGCAATGCGTCATCAC | Cloning the 3’ flanking region of *rac* |
| 3’R | CCCGGGGATCCTCTAGAGATCAAAGACACAAAGACACAAA | Cloning the 3’ flanking region of *rac* |
| yRac-F | GGACAAGCATGGCGGCTGAATCTG | Verifying the*△rac* transformant |
| yRac-R | CAAAGACACAAAGACACAAAGAGGAAGATTTGAAG | Verifying the*△rac* transformant |


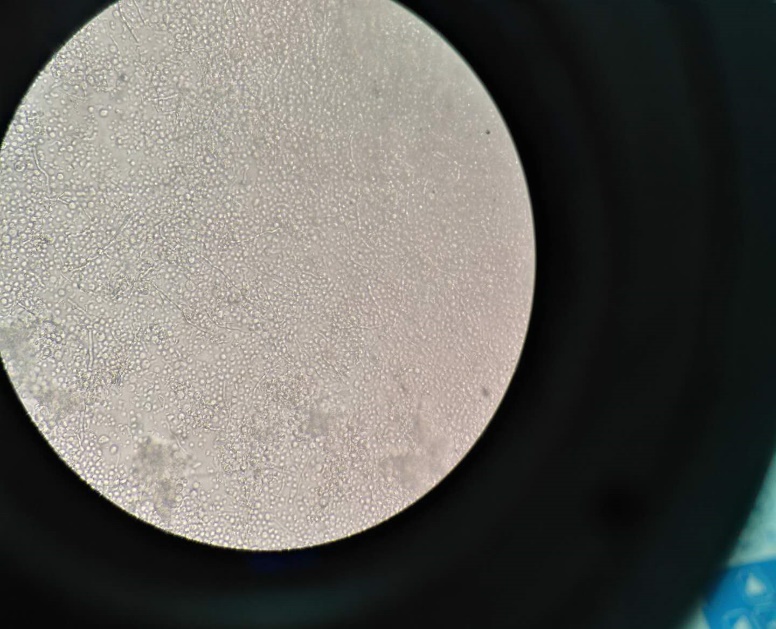


**Fig. S1** Microscopic observation of the protoplasts of *C. purpurea* 3.1003 prepared.


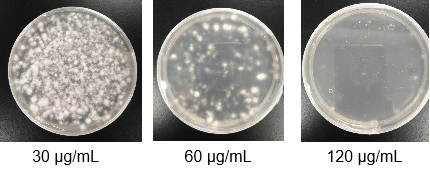


**Fig. S2** Protoplast regeneration of *C. purpurea* 3.1003 under different concentrations of phleomycin.


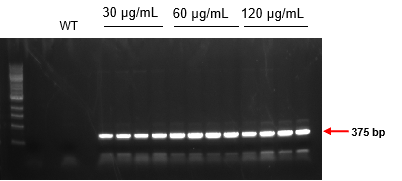


**Fig. S3**  Diagnostic PCR validation of transformants picked out under different stress of phleomycin.


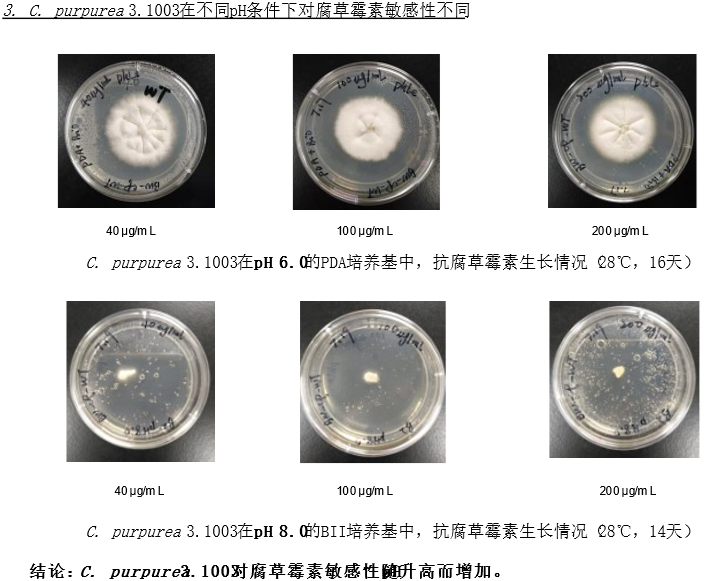


pH 6.0


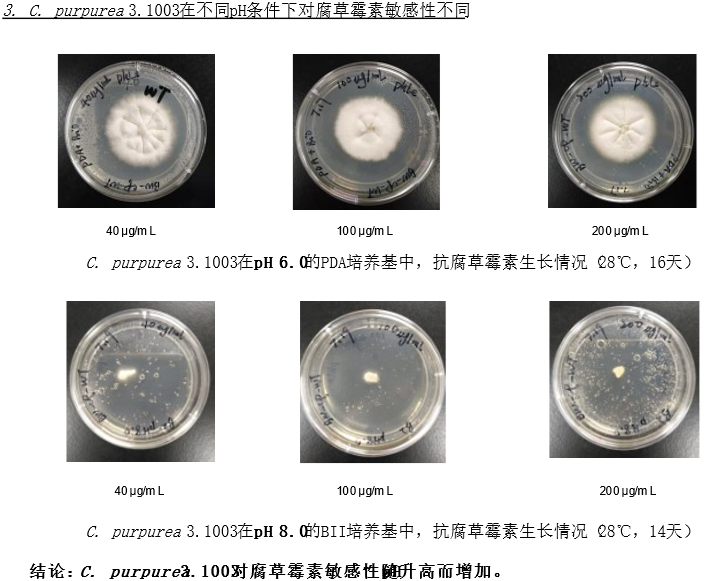


pH 8.0

**Fig. S4** Resistance sensitivity of *C. purpurea* 3.1003 to phleomycin on plates with various pH values.


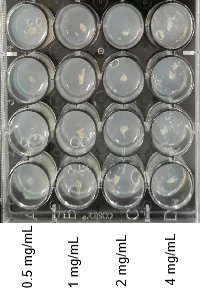

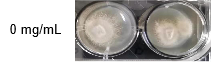


0

0.5 1.0 2.0 4.0

mg/mL

**Fig. S5** Resistance of *C. purpurea* 3.1003 to hygromycin on PDA plates.


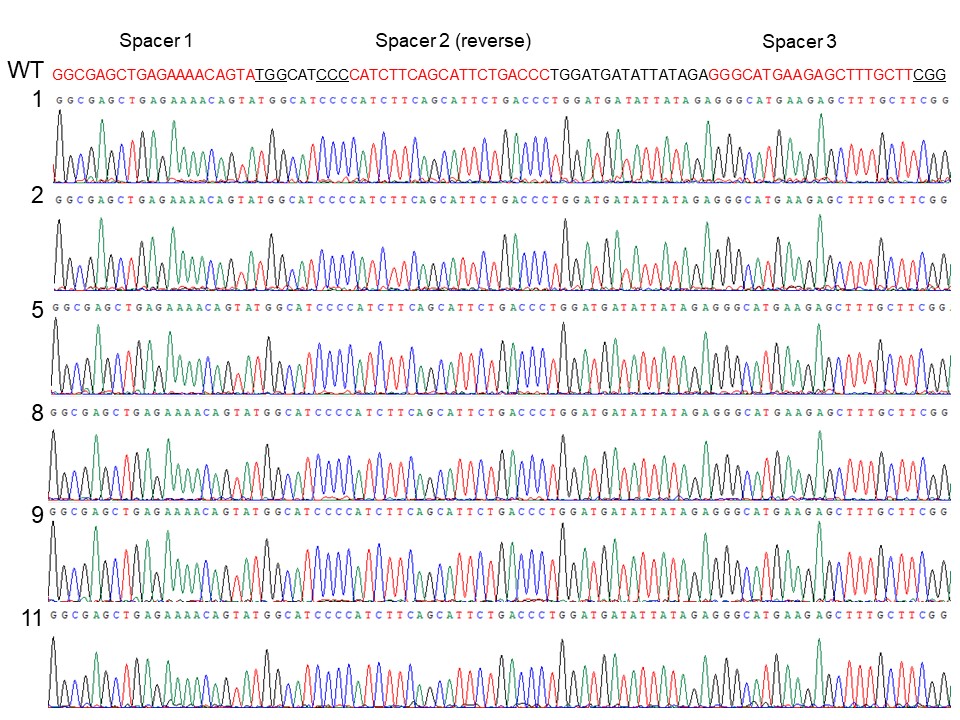


**Fig. S6** Sequences of *ura5* gene of the false positive colonies.

**Fig. S7** Phenotype of *C. purpurea* and *ura5*-deficient mutant on PDA plates with 0.3 mg/mL 5-FOA. Cultivation was done for 4 weeks at 28℃.

**Fig. S8** Putative biosynthetic pathway of ergot alkaloids in *C. purpurea*. The dashed arrow indicates an unresolved step, and the solid-line arrows indicate that the step has been resolved. Ergot alkaloids catalyzed by *easA* in the pathway are marked in red.

**Fig. S9** Analysis of the fermentation products of *C. purpurea* and the Δ*easA* mutant. Three Δ*easA* transformants were tested. Black dotted line region of the HPLC spectrum was zoomed in to show detailed information.
